# Low-Fidelity Prototype of a Sensor-Dependent Interaction Platform: Formative Evaluation With Informal Caregivers of Older Adults With Cognitive Impairment

**DOI:** 10.2196/53402

**Published:** 2024-03-22

**Authors:** Nikita Sharma, Karen Grotenhuijs, J E W C van Gemert-Pijnen, Harri Oinas-Kukkonen, L M A Braakman-Jansen

**Affiliations:** 1 Faculty of Behavioural, Management and Social Sciences University of Twente Enschede Netherlands; 2 Faculty of Information Technology and Electrical Engineering University of Oulu Oulu Finland

**Keywords:** older adult care, informal caregivers, cognitive impairment, sensing solutions, information communication platform, low-fidelity, lo-fi prototype

## Abstract

**Background:**

Unobtrusive sensing technologies developed for monitoring deviant behaviors in older adult care require integration with an interaction platform to facilitate the flow of information between older adults and their caregivers. However, the continuous monitoring capabilities generate a considerable amount of data that must be interpreted, filtered, and personalized before being communicated to the informal caregivers based on their specific care needs and requirements.

**Objective:**

For the effective implementation of unobtrusive sensing solutions (USSs) in the care of older adults with cognitive impairment, we aimed to explore the expectations and preconditions regarding the implementation of USSs from the perspective of informal caregivers. Subsequently, we designed and evaluated a low-fidelity prototype of an interaction platform for its conceptual workflow and usability, incorporating persuasive system design features based on the needs and requirements of informal caregivers.

**Methods:**

Overall, 6 informal caregivers of older adults with cognitive impairment living alone participated in this qualitative interview study. We explored the expectation and preconditions regarding implementation through open-ended questions and conducted a formative evaluation (usability study with a think-aloud approach) to evaluate the conceptual workflow and used persuasive system design features in the interaction platform. Overall, a combination of inductive and thematic analyses was used to analyze the interviews.

**Results:**

The results of this study present both positive and negative outcome expectations regarding the implementation of USSs, highlighting benefits such as objective decision-making and peace of mind and concerns about information overload and the potential substitution of human contact. Strategic information communication agreements between informal and formal caregivers were deemed crucial for the successful implementation of USSs in care. Overall, informal caregivers had a positive experience with the low-fidelity prototype of the interaction platform, particularly valuing the personalization feature.

**Conclusions:**

In conclusion, to achieve successful implementation, a holistic design approach is necessary, and equal consideration should be given to the personalization-privacy paradox to balance users’ needs and privacy.

## Introduction

### Background

The increase in the older adult population (≥65 years) imposes significant challenges on the organization and functioning of the current health care infrastructure worldwide [1]. It demands the active involvement of different stakeholders including informal caregivers, formal caregivers, general practitioners, technology developers, policy makers, and government organizations to maintain continuous care [2]. Primarily, informal caregivers are perceived as responsible for organizing and ensuring on-time care for older adults, which impacts their physical, financial, emotional, and social well-being [3,4]. Moreover, with the emergence of cognitive impairment or comorbidities, the care process becomes more complex and challenging for informal caregivers [5].

To support informal caregivers in delivering on-time care, sensor-based solutions, specifically those that are unobtrusive or device free (ie, do not demand direct involvement or attention from older adults), are being developed [6]. Studies have shown that unobtrusive sensing solutions (USSs) are in demand and appear to be useful among informal caregivers of older adults with cognitive impairment due to their 24/7 monitoring capabilities, which provide real-time insights into the health of care recipients [2,6]. A USS comprises 3 main units: a sensing unit responsible for collecting data from the care recipient, a computing unit responsible for analyzing the obtained sensing data, and a communicating unit that communicates the output of the computing unit to the informal caregivers to enable monitoring at a distance [7].

Over the past decade, there have been notable advancements and successful endeavors to facilitate the development of unobtrusive and ubiquitous sensing technology [8]. For example, Wi-Fi channel state information (CSI; as a sensing unit) can be used for monitoring physical activities (falls, sitting, hand gestures, etc), physiological activities (heart rate and breathing rate), and behaviors (sleeping patterns, personal hygiene, etc) [9-11]. Significant growth in the computing unit through the use of advanced machine learning methods (such as deep neural networks, generative adversarial networks, etc) to improve privacy, reliability (minimizing false alarms), and computing time is also evident [12,13]. However, efforts from the IT domain (communication unit), particularly in the direction of developing and designing interaction platforms adhering to the information communication (IC) needs and requirements of informal caregivers (or other stakeholders), are limited [14]. Designing an interaction platform according to the preferences of informal caregivers can assist in prioritizing and optimizing their care plans, thereby reducing the care (information) load [7].

In our previous multimethod study encompassing a survey and interviews with informal caregivers of older adults with cognitive impairment, diverse needs and requirements for the following 4 distinct care scenarios were explored: falls, nocturnal unrest, agitation, and normal daily life [7]. The findings indicated varying information needs regarding the mode, content, timing, and stakeholders involved, contingent upon the care scenario. In addition, these needs were observed to be influenced by the personal circumstances of caregivers and care recipients and the progression of illness in care recipients. Furthermore, to facilitate the designing of an interaction platform (ie, this study) persuasive system design (PSD) features, namely reduction, tailoring, personalization, reminders, suggestions, trustworthiness, and social learning, were identified for the involved care scenarios. One of the limitations that we observed was the lack of proper understanding of USSs among informal caregivers. Due to the technical novelty of the solution, informal caregivers perceived it as a black box, potentially introducing biases in their responses regarding its usefulness and expectations.

### Objective

Building upon the findings from our previous study [7], the objectives of this study were two-fold: (1) to explore the expectations (positive and negative) and preconditions regarding implementing USSs in the care of older adults with cognitive impairment from the perspective of informal caregivers after showing them a video prototype of the solution and (2) to design and evaluate a clickable, low-fidelity (lo-fi) prototype of a sensor-dependent interaction platform, incorporating the identified PSD features regarding fall, agitation, and normal daily life care scenarios with informal caregivers.

## Methods

### Ethical Considerations

The Ethics Committee of the Behavioral, Management, and Social Sciences department at the University of Twente formally approved the execution of this study (request number 230141). Before engaging in the surveys and interviews, participants received both oral and written description elucidating the study’s objectives, methodologies, data collection procedures, storage protocols, and the intended use of the collected data. Subsequently, each participant provided a signed consent form. The participants were also free to withdraw from the study at any stage if they felt uncomfortable.

Finally, the participants were offered a small honorarium as a token of appreciation for their valuable time and contributions.

### Study Design: Participatory Development

**Figure 1:**
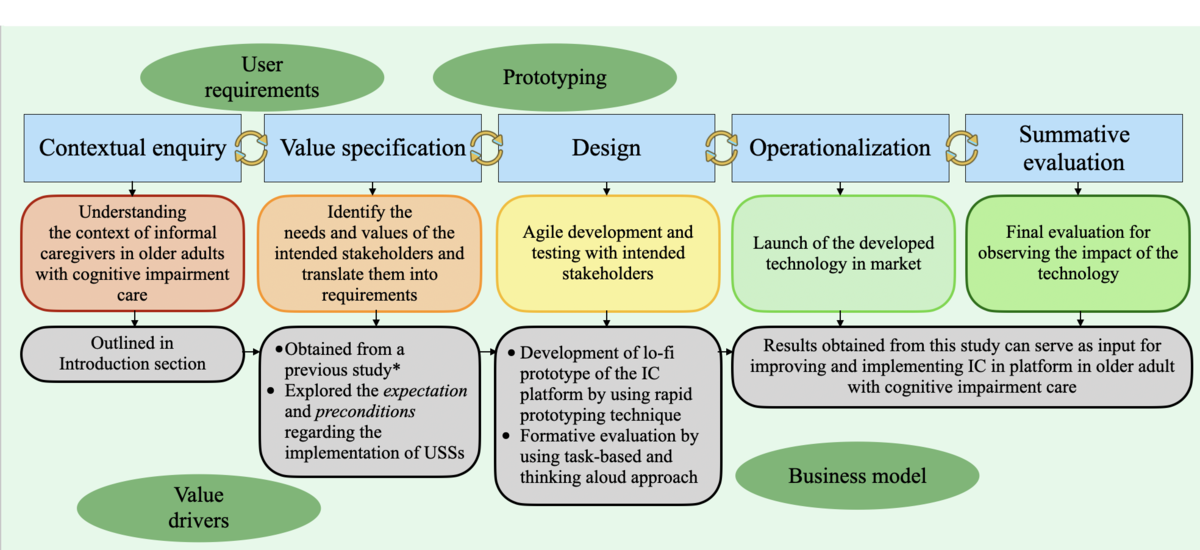
The Center for eHealth Research and Disease Management framework. IC: information communication; lo-fi: low-fidelity; USS: unobtrusive sensing solution. *The study by Sharma et al [7].

The study followed the Center for eHealth Research and Disease Management (CeHRes) road map to create a sensor-dependent interaction platform that can communicate the information obtained by USSs to informal caregivers of older adults with cognitive impairment [15]. The CeHRes road map fosters progress toward context-aware sensing and computing by offering early feedback regarding users’ needs and requirements to the designers and developers [15]. For instance, if informal caregivers prefer insights into emergencies only, the algorithm can be trained and optimized accordingly to provide relevant data, avoiding computing overload for the system and information overload for the caregivers. The framework encompasses 5 distinct but intertwined phases: contextual inquiry, value specification, design, operationalization, and summative evaluation (Figure 1 [7]). The description of these phases, along with their relevance to this study, is provided in Textbox 1.

**Description of Center for eHealth Research and Disease Management (CeHRes) phases along with their relevance this study.**

**CeHRes phases and description of relevance to this study**

- Contextual inquiry
  This phase helped in building an understanding of the prospective users (informal caregivers) and their context (care of older adults with cognitive impairment)
  The understanding of experiences, expectations, and usefulness of unobtrusive sensing solutions (USSs) among informal caregivers of older adults with cognitive impairment were obtained from a previous study [7]
  To further advance the findings, we dwelled deeper into informal caregivers’ expectations and preconditions regarding the implementation of USSs after providing them with more detailed information about the functioning and potential benefits of using USSs in the care of older adults with cognitive impairment
- Value specification
  This phase helped in identifying the needs and values that are important for the intended stakeholders (informal caregivers), which can be translated into the requirements later [15]
  Information communication design requirements regarding different care scenarios (fall, agitation, and normal daily life) obtained in a previous mixed methods study [7] were used to develop the interaction platform
- Design
  The primary focus of this study lied within the design phase that involves the agile development and testing of the interaction platform
  A low-fidelity prototype was developed by using the rapid prototyping technique by the involved researchers’ team [16,17]
  The prototype was subjected to formative evaluation with informal caregivers, with specific emphasis on evaluating the conceptual workflow and used persuasive system design features
  A task-based study design [18,19] in conjunction with a think-aloud approach [20] was used for this study
- Operationalization and summative evaluation
  These phases will be activated when the technology is launched into the market. The results obtained from this study can serve as valuable inputs for these phases, ensuring that the final product meets the needs and requirements of informal caregivers of older adults with cognitive impairment

### Participants

Participants were recruited from an already existing pool of candidates who had previously been involved in a similar study [7]. While previous experience with digital care technology was not a prerequisite for informal caregivers, all of them were users of the Caren Platform (a digital care platform; Caren [NEDAP]), implying that they had default experience with digital care technology [21]. Informal caregivers were approached for participation in this study via email. The participants received an invitation, along with an information letter describing the study’s purposes, procedures, and the researcher’s contact information. When an informal caregiver expressed willingness to participate, they were screened based on the following inclusion criteria: (1) providing unpaid care to a person with cognitive impairment who is a relative, friend, or someone else within their personal circle; (2) the person with cognitive impairment is aged ≥65 years; and (3) the person with cognitive impairment lives alone at home. Subsequently, an appointment for the evaluation session was scheduled with the researcher.

### Materials: Designing the Lo-Fi Prototype of the Interaction Platform

#### Video Prototype

Given the novelty of USSs, a lack of awareness among informal caregivers about their working and implementation was observed. Therefore, to educate informal caregivers, a video prototype demonstrating the working, system architecture, and benefits of USSs in the care of older adults with cognitive impairment was created. While the video was largely inspired by previous studies that used Wi-Fi CSI as a technology in USSs for recognizing older adult activities [11], some brainstorming sessions with the research team (composed of eHealth researchers, experts, technology developers, and designers) also occurred to align it with the use case of care of older adults with cognitive impairment.

Overall, the video depicted 3 units of USSs, namely, sensing, computing, and communicating units. The “sensing unit” of the solution showed the working (how) and the manner of data collection (what) through Wi-Fi CSI (as an unobtrusive sensing technology). The “computing unit” of the solution presented the use of artificial intelligence (AI) algorithms for analyzing the collected data. In the “communicating unit” of the solution, the channel for communicating the computed information to the caregivers was presented. The video provides examples of 3 different care scenarios, namely fall incident, agitated behavior, nocturnal unrest, and normal daily life (drinking activity).

**Figure 2:**
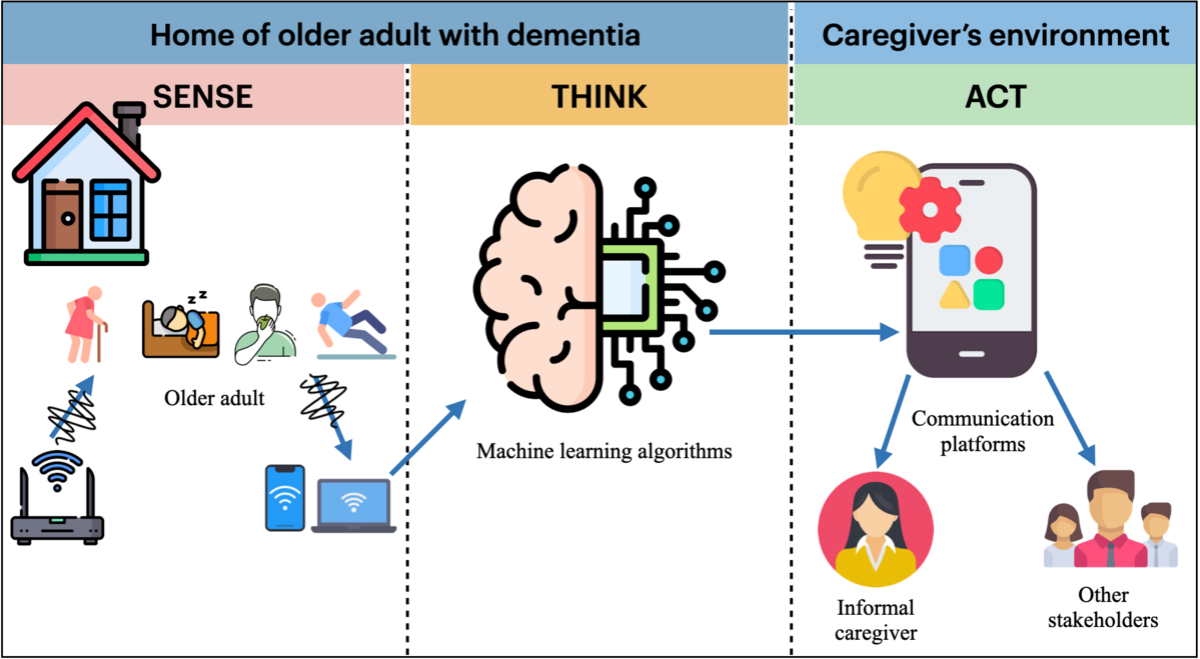
Simplified overview of the system architecture of unobtrusive sensing solutions.

To make the video footage realistic, it was recorded in the eHealth house at the University of Twente, the Netherlands [22]. The video had a Dutch voice-over with English subtitles, given that informal caregivers were comfortable with Dutch. The video has a total duration of 3.5 minutes. It was presented to the participants at the start of the interview sessions to ensure that they had the necessary information to answer the questions posed in the interview, thereby promoting more informed responses. Figure 2 shows a simplified overview of the system architecture (as conveyed in the video) of the intended USSs.

#### Lo-Fi Prototype

##### Overview

We designed the lo-fi prototype of the interaction platform using Figma software [22]. As the Wi-Fi CSI system is in the early development phase (Technology Readiness Level 2/4) [7,23], a lo-fi prototype was chosen to gain initial insights from informal caregivers about the communication unit, showcasing the conceptual workflow and main functionalities of the interaction platform. Note that this interaction platform does not intend to change the behavior of the informal caregivers but requires persuasion to form (ie, F outcome) or alter (ie, A outcome) the behavior of informal caregivers for complying (ie, C change) with the information communicated (outcome: F and A; change: C) [24].

The findings from previous studies [2,7,25] were used to design the lo-fi prototype of the interaction platform. The studies by Wrede et al [2,25] demonstrate the value of USSs in continuous and objective monitoring, leading to timely interventions. In particular, informal caregivers found USSs to be helpful in clearly classifying the care scenarios as urgent, nonurgent, and future risk. Further exploration in a multimethod study by Sharma et al [7] (comprising a survey [n=464] and interviews [n=10]) revealed divergent IC needs in different care scenarios (fall, nocturnal unrest, agitation, and normal daily), including mode, content, timing, intended users, feedback to the system for self-learning, and dialogue support. Furthermore, the study also identified 7 PSD features: 3 from primary task support (reduction, tailoring, and personalization), 2 from dialogue support (reminders and suggestions), 1 from system credibility (trustworthiness), and 1 from social support (social learning) for designing the interaction platform. On the basis of these findings, the conceptual workflow of the interaction platform and user interfaces for 3 care scenarios, namely, fall incident, agitated behavior, and normal daily life activities were designed. In addition to these features, a system verifiability feature was added to assess its necessity or impact on the interaction platform [26]. Table 1 presents the used PSD features, their interpreted meaning, and their application in the lo-fi prototype. The following sections provide details regarding the design of the conceptual workflow and user interfaces.

**Table 1:** Persuasive system design (PSD) features used to design the low-fidelity (lo-fi) prototype.

PSD category, feature, and meaning			Application in the lo-fi prototype
**Primary task support**			
	**Personalization**		
		Providing personalized content	Option to personalize IC^a^ based on the individual needs and requirements
	**Reduction**		
		Reducing complex tasks into smaller tasks	Immediate notifications during emergencies and real-time updates on the home screen
	**Tailoring**		
		Providing information tailored to the user’s needs	Tailored reports and notifications according to the needs of the recipient
**Dialogue support**			
	**Reminder**		
		Reminding users about the target behavior	Reminder for unattended emergency call
	**Suggestion**		
		Offering suggestions to facilitate behavior	Customized care suggestions for informal caregivers in different care situations
**Social support**			
	**Social learning**		
		Learning from the experiences and behavior of others	Page for sharing experiences where the informal caregivers can read and react to the experiences of others
**System credibility**			
	**Trustworthiness**		
		Providing reliable information	Reliability percentage indicator and provision to provide feedback to the system
	**Verifiability**		
		Providing evidence to validate the accuracy	Caregiver support and communication page that helps informal caregivers discuss the care plans and provides an option to verify the system’s predictions

^a^IC: information communication.

##### Design of the Conceptual Workflow

**Figure 3:**
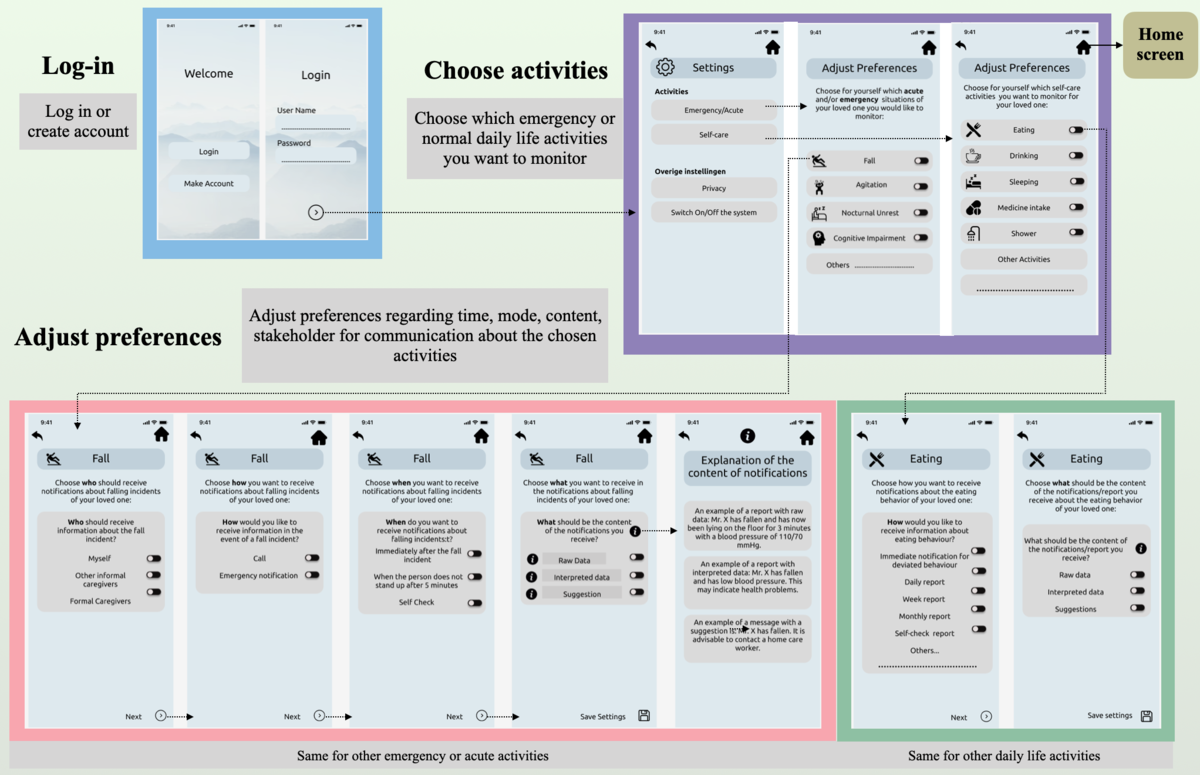
Conceptual workflow of the interaction platform.

The conceptual workflow of the interaction platform is illustrated in Figure 3. This workflow reflects the logical flow of the interaction platform when personalizing the IC options. It starts from the log-in page, followed by choosing the preferred activities for monitoring; adjusting the communication preferences for the chosen activities; and the home screen, where multiple functionalities of the interaction platform can be checked or adjusted. Note that the feature of choosing the activities to be monitored and adjusting the preference is attributed to the *personalization* feature of the PSD model.

##### Design of the User Interfaces

**Figure 4:**
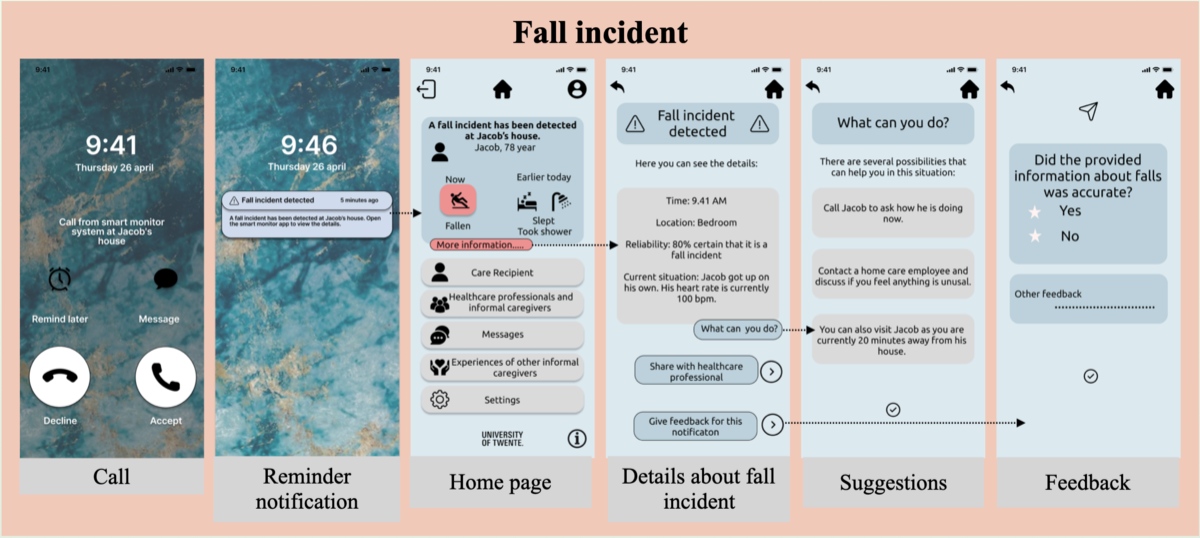
Interfaces for fall scenario.

User interfaces for fall, agitation, normal daily life, and home screen were designed. As falling is an emergency scenario, informal caregivers expect to receive a direct call (*reduction feature*), and if they do not respond within 5 minutes, they expect a reminder notification (*reminder feature*) in their preferred content style (raw, interpreted, or suggestions). Furthermore, the details of the fall incident such as time, location, system’s confidence in prediction, and current state of the care recipient were made accessible to the informal caregivers. In addition, to support informal caregivers, options to obtain suggestions from the system regarding what to do and when to act *(suggestion feature)* and to directly communicate with formal caregivers were also provided. Finally, as informal caregivers desire a trustable system (with minimum false alarms), an option to provide feedback to the system about its predictions to enable self-learning was also added (*trustworthiness feature*). Figure 4 illustrates the interfaces for the fall scenario.

**Figure 5:**
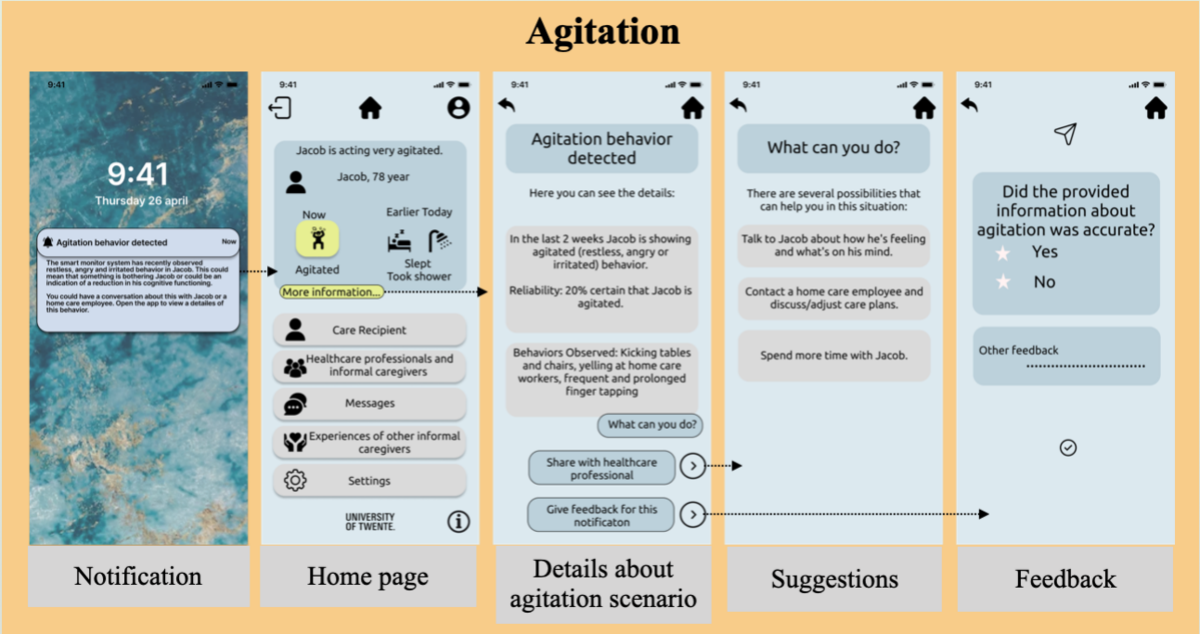
Interfaces for agitation scenario.

On the other hand, as agitation is an acute scenario, informal caregivers expect the system to monitor it for a few weeks and share a report tailored to the concerned stakeholders, that is, themselves or formal caregivers (*tailoring feature*). Interfaces depicting notification (in the preferred content style), details about agitation behavior (duration, system’s confidence in prediction, and other observations), suggestions from the system (*suggestion feature*), the possibility to share the report with formal caregivers, and an option to provide feedback to the system for its predictions were designed (*trustworthiness feature*). Figure 5 illustrates the interfaces for the agitation scenario. The user interface for normal daily life (Figure 3) presented multiple self-care activities (eating, drinking, showering, etc). The informal caregivers can adjust their preferences for the content, frequency, and depth of information they want to receive regarding the selected activity.

**Figure 6:**
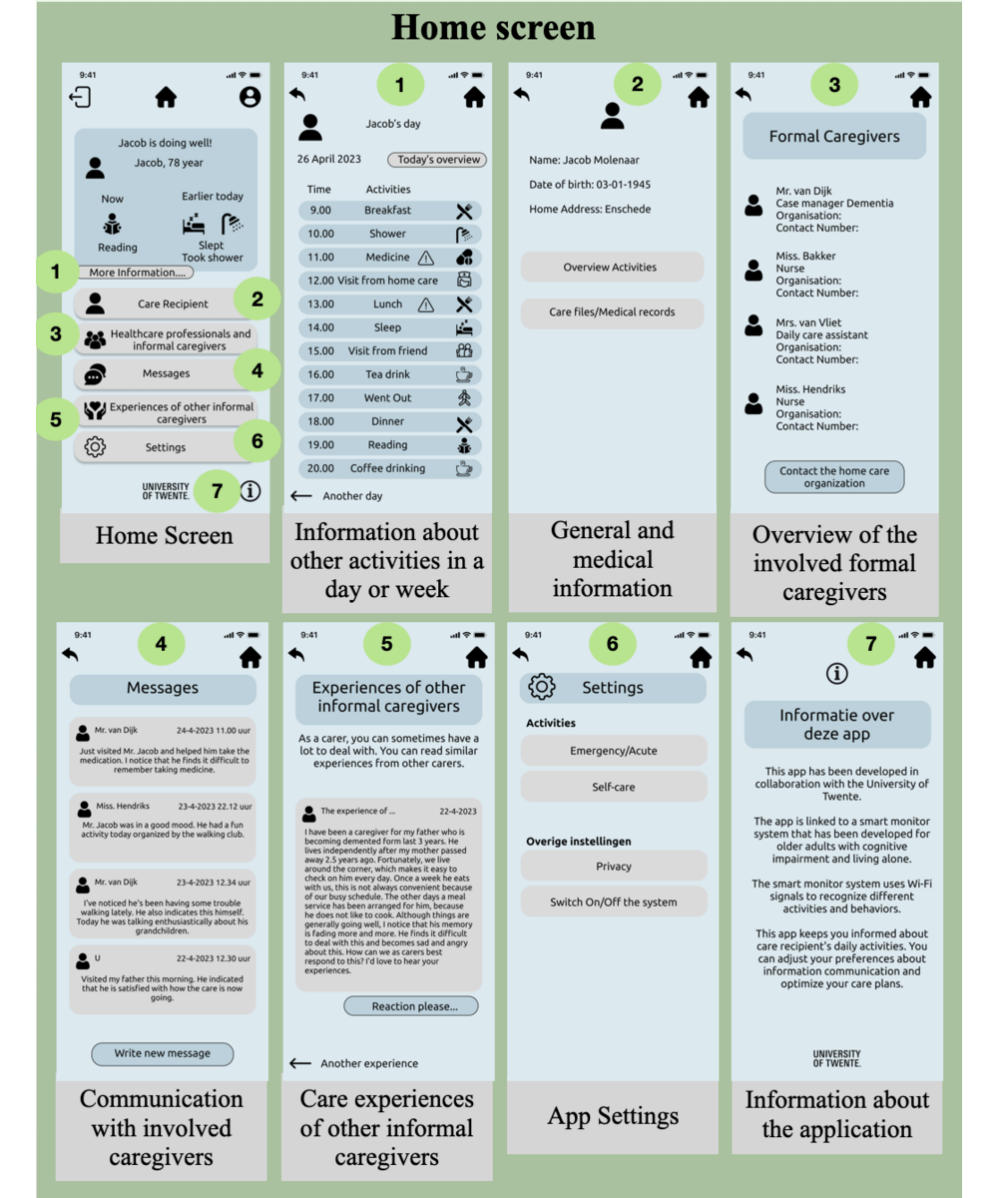
Interfaces for home screen.

In general, informal caregivers demand a centralized care approach, that is, the possibility to access all the relevant care information such as general information, medical information, and communications with other stakeholders in 1 platform [7]. Thus, consistent with this requirement, the home screen contained the following functionalities: observing the present and past situation of the care recipient (*reduction*), obtaining more detailed information or reports about daily activities, general and medical information about the care recipient (verifiability), an overview of the involved formal and informal caregivers, communication options with the involved formal caregivers, and system credibility (Figure 6). In addition, an option to read the care experiences shared by other caregivers as a part of the *social learning feature* of the PSD model was added. Finally, options for app settings (adjusting preferences) and information about the organization or team developing the app to show *system credibility* (*real-world feel*) were also added.

### Procedure

The semistructured interviews were conducted via Microsoft Teams with 33% (2/6) of the informal caregivers and in person with 67% (4/6) of the caregivers, based on their preference. The interview guide (Multimedia Appendix 1) was used and consisted of the following sections: (1) introduction (goals, procedures, and informed consent), (2) background information obtained from the participant, (3) video prototype to explore the expectations (positive and negative) and preconditions, (4) formative evaluation of user interfaces, and (5) closing remarks. Upon watching the video, participants were asked whether they had any further questions regarding the systems, and the questions were clarified. This video and explanation were important because, due to the novelty of USSs, informal caregivers are not very aware of this concept or type of sensing solution. Then, their expectations and preconditions regarding implementation were discussed in depth.

Thereafter, a formative evaluation (using a task-based and think-aloud approach) of the designed interaction platform was conducted using 5 tasks (Table 2). In task 1, the informal caregivers were asked to choose the emergency or acute situations of their care recipient that they want to monitor, followed by adjusting the IC preferences for the chosen activities. Similarly, in task 2, the informal caregivers were asked to choose and adjust their IC preferences for the daily life (self-care) activities of the care recipient that they want to monitor in the long term. Here, the use of PSD feature personalization was evaluated. Furthermore, for tasks 3 and 4, a possible sequence of actions in the fall and agitation scenarios was evaluated. Specifically, the ability of the platform to immediately call or notify informal caregivers (reduction), send reminder notifications in case they do not respond (reminder), provide suggestions to support the informal caregivers (suggestion), and maintain a transparent link between the system and caregiver by providing the predication percentage (trustworthiness) were assessed.

**Table 2:** Tasks used to evaluate the conceptual workflow and persuasive system design features of the interaction platform.

Task description	Feature added and evaluated	Value associated
Task 1: Choose emergency activities and adjust preferences for the chosen activities Task 2: Choose self-care activities and adjust preferences for the chosen activities	Personalization	Every care scenario is different, and thus, informal caregivers should be able to choose which activity they want to monitor (for both emergency and daily life). Furthermore, they should also be able to adjust the preference regarding IC^a^ for the chosen activities. Informal caregivers need the flexibility to select and monitor specific activities based on the care scenario. They should also have the option to customize their preferences for IC related to the chosen activities [7,25].
Task 3: Suppose a fall incident occurred in the home of your care recipient	Reduction Reminder Suggestion Trustworthiness	During emergencies, informal caregivers expect the following: direct calls or reminders if they are unable to answer, trustworthy and accurate information, and suggestions to ensure timely and appropriate actions [7]
Task 4: Suppose your care recipient is experiencing agitation	Reduction Tailoring Suggestion Trustworthiness	During acute scenarios such as agitation, informal caregivers expect the following: notification and long-term reports that can be shared with formal caregivers and trustworthy and accurate information along with suggestions to support the care recipient in the right manner [7]
Task 5: Explore the features of the home screen	Reduction Verifiability Social learning System credibility	Informal caregivers desire a centralized care platform, where they can find important care elements immediately, eg, quick or detailed overview of the activities, access to medical records, and connection with caregivers [7]

^a^IC: information communication.

Finally, in task 5, the informal caregivers were asked to explore the home screen to check whether it satisfies their requirement of a centralized care platform and present evidence to validate the provided information (verifiability). They were encouraged to identify, suggest, and reason the functionalities that help them in improving the caregiving process. The sessions were conducted in Dutch by a native Dutch speaker and were audio recorded to facilitate analysis. The duration of each session was approximately 60 minutes. On the basis of the feedback obtained from the first 4 sessions, slight improvements in the design were made and further evaluated in the last 2 sessions.

### Data Analysis

The recordings were transcribed verbatim by using the description software, Amberscript. Qualitative analysis was performed by using Atlas.ti [27]. A thematic analysis was performed, based on the six steps by Braun and Clarke [28]: (1) familiarizing with the data, (2) generating initial codes, (3) searching for themes, (4) reviewing themes, (5) defining and naming themes, and (6) producing the report. The transcripts were coded using a mixed inductive and deductive approach. An inductive approach was used for exploring the expectations and preconditions, whereas an inductive-deductive approach was used for analyzing the experiences with PSD features. All the transcripts were read by both researchers, NS and KG, in English and Dutch, respectively. Overall, the joint probability of agreement was 75%, followed by an in-depth discussion until consensus was reached regarding all the defined themes.

## Results

This section is divided into 2 parts: first, the results related to the expectations and preconditions regarding implementation are presented, and second, the results corresponding to the formative evaluation of the lo-fi prototype including PSD features are presented.

### Demographics

A total of 6 informal caregivers (mean age 58.7, SD 2.87 years) of older adults (mean age 85.7, SD 4.18 years) living alone participated in the study. Among the 6 participants, 4 (67%) were women and 2 (33%) were men. All informal caregivers (6/6, 100%) were children of the care recipient and were the primary informal caregiver. All care recipients (6/6, 100%) were living alone. Half of the care recipients (3/6, 50%) had Alzheimer disease, and the other half (3/6, 50%) had cognitive impairment due to other causes or no official dementia diagnosis. All informal caregivers (6/6, 100%) have used technology previously in the care provision, differing from using communication platforms (Caren platform) or medication dispensers to personal alarm and monitoring systems. They have been providing care for at least the past 2 years, and their care hours ranged from 1.5 to 15 hours. Table 3 provides an overview of the characteristics of the interview participants.

**Table 3:** Sociodemographic characteristics of informal caregivers.

Participant number	Sex	Age of the informal caregiver (years)	Age of the care recipient (years)	Experience in providing informal care (years)	Time spent on informal care (hours/week)	Distance from or time needed to reach the care recipient
1	Female	61	87	2	5	30 minutes
2	Female	59	88	2-3	1.5	2 hours
3	Female	60	86	3	8	Next door
4	Female	53	79	2	10-15	1.5-2 km
5	Male	60	91	20	12	45 minutes
6	Male	59	83	5	2	20 minutes

### Expectations and Preconditions

The expectations and preconditions regarding implementation are presented as three broad themes: (1) positive outcome expectations, (2) negative outcome expectations, and (3) preconditions for implementation.

#### Positive Outcome Expectations

##### Objective Decision-Making

The informal caregivers indicated that USSs could contribute toward making objective decisions regarding the care of their loved ones. Instead of relying solely on observations of both informal and formal caregivers or on what the care recipient mentions themselves, the system can provide the involved informal and professional caregivers with more objective and in-depth monitoring information. According to informal caregivers, this information not only enables prompt diagnosis of underlying health conditions but also facilitates objective communication among the professional caregivers, care recipient, and themselves. This fosters a shared understanding of the care situation, consensus about the provision of care, and better coordination regarding the response to the (emergency) situations:

It provides the facts, so what she herself isn’t mentioning yet, but what is actually already there, that could be beneficial to support her, to make better choices and to better understand what is going on.

Participant 2

It provides monitoring information, for example, we are now at a stalemate with my father, he should have more help and we need to request that, but he doesn’t want that because he believes he can still manage. It [the monitoring data] can prove that we are right, but it can also prove if he is right. If he is right, then we’ll have some peace for a while, so it indicates such things.

Participant 4

##### Safer Environments for Independent Living

Informal caregivers expected USSs to contribute to the feeling of safety of their loved one; they believe that the system will notice when a safety risk occurs. Moreover, it was mentioned that the system could give insight into whether it is safe for older adults to live independently at home. All informal caregivers (6/6, 100%) mentioned being interested in receiving information about safety matters, for instance, if the door has been opened or if the gas is on:

She [mother of informal caregiver] will feel safer. Her desire is to continue living at home for as long as possible, but she has concerns about it, like: “yes, I am alone and if something happens to me, well, what should I do then?” And this is a system that detects it [a fall] without her having to do anything. So, if she feels safer, she will also feel calmer, which has an impact on her dementia symptoms.

Participant 1

##### Providing Peace of Mind to Informal Caregivers

Some informal caregivers indicated that they expect USSs to contribute to their peace of mind and probably to the peace of mind of their care recipient also. They find it reassuring that the system acts as a safety net and alerts them or the care professionals when there is an emergency situation. Furthermore, they indicated that the system could confirm the well-being of their loved one, whereas without such as system, there would be uncertainty and doubt about the situation, and they might be unnecessarily worried about their loved one:

It brings peace of mind. It provides, like, you can’t fully rely on the technology, but knowing that you have an additional safety net, that you are a bit more at ease, and also for the person involved it helps.

Participant 3

I only see reassurance, you know, you receive, you know that everything is fine, but you receive a confirmation that it is indeed going well.

Participant 5

##### Stimulating Meaningful Conversations

A few informal caregivers indicated that if USSs can collect care-related information, they might be able to spend more meaningful time (personal conversations) with their loved ones. This is because the care component is important and requires a lot of attention, and they overlook the personal or relational aspect, thus impacting their relationship with the care recipient:

Because it’s not constantly asking “how are you doing,” there is an additional aspect behind it. Yes, you still have to keep asking, but it’s more about showing interest in the person rather than focusing solely on the care component. So, I think there’s more room for the human aspect rather than just the caregiving aspect.

Participant 3

It can help in relational aspect, I would really appreciate that, because I miss the conversations with my mother, there is always that caregiving component that comes in-between.

Participant 3

##### Negative Outcome Expectations

###### Information Overload

A few informal caregivers also expressed concerns about the possibility of information overload from USSs. They mentioned that the continuous availability of information about the care recipient, enabled by USSs, might lead them to constantly check and monitor every aspect of their loved one’s situation. In addition, informal caregivers highlighted that receiving notifications might trigger panic and worry, particularly if they are unable to respond immediately, even after being aware of it:

At some point, you want to know everything. Especially if you’re worried, then it’s nice to be able to see a lot, yet you can’t do anything with it.

Participant 2

If I look at myself...I think if I receive such a notification [emergency] then the first reaction is panic, okay that is a strong word, but as I already said: I work in healthcare myself, I see the most terrible things, that doesn’t affect me. But when it concerns your own parents, it immediately causes stress.

Participant 4

###### Feeling Obliged to Undertake Action

The informal caregivers also mentioned that once they are aware of their loved one’s situation, they cannot ignore the situation and they feel obliged to undertake actions according to the information provided by USSs. However, sometimes, it is simply not possible to take action immediately due to physical distance or other factors. However, some participants indicated not having the urge to immediately act upon the data or being able to filter important information; they suggested it might be problematic for other informal caregivers:

If I see worrying things, then I literally and figuratively have to go there, if I see it, then I have to go there: normally, you wouldn’t, or quickly call, but now you see it, so you feel compelled to go there...

Participant 4

So for my situation that [information overload] won’t happen so quickly. For my sister, it might be a bigger struggle, as she is less able to distance herself from the situation as it is. I think when she receives detailed information from the system, she may feel the need to intervene, whereas I have less trouble with that.

Participant 6

###### Substitution of Human Contact

Although not all informal caregivers expect the system to substitute the human contact of their care recipient, some of them perceived this as a risk from using USSs. They suggested that if USSs are capable of providing comprehensive insights into the health of the care recipient, it could potentially result in reduced or no visits from professional care staff. This is concerning, considering the already existing scarcity of professional caregivers:

It’s simply impossible to find enough staff, and apparently the situation is even worse in home care. So, if you’re going to develop technology to do more, with fewer people, to be more efficient, it means there will be less human contact, and that means less home care visits for my father, while on the other hand, he’s already experiencing so much loneliness.

Participant 4

I find it a risk that people retrieve all their information from this system and they might start thinking they no longer need the contact moment, while it is actually so important.

Participant 1

##### Preconditions for Implementation

###### Shared Decision-Making

According to informal caregivers, it is necessary to discuss and reach an agreement together with professional caregivers about IC, including which activities should be monitored, what communication strategy should be used, who should receive and respond to the information, and what should be the content of the information. This would be necessary to prevent unclarities, unfulfilled expectations, and unaddressed notifications or follow-ups, as it could otherwise potentially hinder the effectiveness of care provision:

You can benefit a lot from it [USSs] together and I think if you don’t do this together, everyone can get a lot of trouble from it. That’s not what you want.

Participant 1

I would never fill it [the settings of the system] in alone, I would really do that together with other professional caregivers or informal caregivers. I think you should all agree with each other about how you fill this in and what you expect and so on...this would be a nice moment to put our heads together and make a choice together.

Participant 1

It could be that you alert three parties simultaneously and one thinks, “hold on, I won’t do anything because the other two will take care of it,” and everyone assumes that of themselves. And then, nobody responds...

Participant 6

###### USS as a Supportive Tool

The informal caregivers indicated that it is important to perceive USSs as a supportive tool rather than a tool to replace the human component in care:

They [persons with cognitive impairment] actually require people around them to be present. It’s better for them, otherwise, they will completely withdraw. Human interaction, maintaining contact with others is extremely important. So that aspect should be preserved. The system should not result in less human contact, as that would further distance individuals with dementia.

Participant 1

However, they expected the solution to provide concrete data to facilitate the conversations about and interpretation of the situation together with formal caregivers:

Cognitive decline happens slowly and there are some things that we [informal caregivers] can’t point out. Now it [wandering in the house] is starting to happen more and more. Such raw data can be important for such situations, especially if you have to go to the neurologist or something, then you can do a lot of things.

Participant 4

It was also mentioned that care decisions should not solely be based on the information provided by the system. Instead, it is imperative to engage in discussions with professional caregivers before making definitive decisions:

So it’s a support system and it shouldn’t take over the analysis of the situation. It may give the numbers, but if on that basis it is said of oh, she [care recipient] only needs so much more care time, or this is no longer necessary since she can still handle this task herself. Yes, then we’re going the wrong way.

Participant 2

### Informal Caregivers’ Experiences With PSD Features and the User Interface Prototype

#### Overview

In general, the informal caregivers indicated being quite positive about the user interface prototype. Most of the screens were reported to be clear and understandable. However, there were also some negative experiences and suggestions for improvement. Overall, the experiences of informal caregivers are presented as three main themes: (1) positive experiences, (2) mixed experiences, and (3) suggestions for improvement.

#### Positive Experiences

##### Personalization: Options to Customize Settings

All informal caregivers (6/6, 100%) had a positive experience with the flexibility (not a one-time setup) of adjusting the interaction platform settings to accommodate their dynamic care needs, thereby improving the quality of life for care recipients. Specifically, they appreciated being able to personalize the settings based on their individual circumstances, the evolving condition of their care recipient, and the monitoring scenario at hand (such as emergencies or self-care activities):

I think this is a good thing. The more you can adjust it to fit your and well in this case my father’s needs and lifestyle, the quality of care can be improved.

Participant 4

We’ll do everything first [make all the settings], and then I’ll figure it out, or change it later. It is nice that I could still make adjustments later on, so that it’s not a one-time set-up.

Participant 3

That depends; do I live next door, or close by, then it might be sufficient to be the only one being notified. But this should be available, like imagine I’m away for a weekend. The other informal caregivers will temporarily take over, then I will adjust whether or not someone is available. And the professional caregivers should also receive that notification. So, I would like to have this screen [settings] flexible, so that you can set it individually, per day or per time.

Participant 5

Well, it’s already quite intuitive. It is good that you can click through quickly on different options within each activity and it is indeed stored for future usage. Also, it is nice that you can always come back and adjust things later if needed.

Participant 6

##### Tailoring: Provide Information According to the Stakeholder

Tailoring alerts, notifications, and reports based on the intended recipients (formal or informal caregivers) were found to be valuable in the development of the interaction platform. Informal caregivers felt that a formal caregiver may require different information compared to an informal caregiver:

The information to professionals should be sent as per their needs. Of course, it will be very different from what informal caregivers need.

Participant 2

For example, the raw data obtained from the sensors could provide more meaningful information to the formal caregivers, whereas they found the interpreted data to be sufficient for themselves: 

The raw data is more useful for the healthcare professional than data which is already interpreted by the system. I don’t want raw data because that won’t help me, so then I would go for interpreted data.

Participant 3

##### Reduction: Being Informed About the Situation Directly

The reduction feature was used in 2 ways: to receive direct calls or notifications in emergency situations, and to provide a quick overview of the current and past activities throughout the day on the home screen. In emergency scenarios such as falls, informal caregivers found the system-generated alerts (via quick calls or notifications) to be valuable, as they have the potential to streamline the communication and facilitate on-time care. This automated approach eliminated the need for caregivers to contemplate whom to contact and bypassed potential delays when reaching out to formal caregivers:

It is about on-time care. I think, if something happens, what do you need to do? Whom should you call to organize care quickly? There must be logical thinking behind it and the system can do it quickly. [Participant 3] 

What I find important is that there is an alarm service-like solution, but initially it could be directed straight to the caregiver, a direct signal from the system saying: “here we see a deviation, this is what the system, the technology detects and intervention may be required here” or “we see a fall, immediate intervention in necessary.”

Participant 6

Furthermore, all informal caregivers (6/6, 100%) found the possibility to glance at the current and past activities in the day on the home screen to be convenient:

I find this [home screen] quite clear now, that you can see which activity has already been performed earlier today, but also what is happening at the moment. This is really nice, and at the top, okay so is the situation at the moment.

Participant 1

Yes, I think this is fantastic, I must say. Specifically, the fact that you do indeed see an interpretation of the situation that the system has apparently determined and everything goes well.

Participant 6

The informal caregivers were also positive about the functionalities of sending messages, finding contact information about caregivers, and connecting to an electronic client dossier as these would address the issue of having to use multiple systems:

I think it is always desirable to have everything in one place and not having to deal with various different systems again.

Participant 2

##### Trustworthiness: Insight Into the Reliability of the Information Provided in the System

Most informal caregivers (4/6, 66%) indicated being positive about the system providing a reliability percentage regarding the notification and information. According to them, a reliability score increases their trustworthiness toward the system:

It is still a technology, sometimes false alarms may occur, for example, when she has dropped something and trying to pick it up. Then, that's okay, that system indicates it is reliability percent. I actually like it. It points towards the trustworthiness of the system and also indicates that at times it can miss classify some things.

Participant 2

When the reliability percentage is high, they sense the urgency and seriousness of the situations and were compelled to take required actions:

A reliability of 80 percent, yes, that did something with me...I thought that I should really take this seriously, like really seriously.

Participant 1

On the other hand, when the confidence percentage was low, informal caregivers might be slightly relaxed, but they still wanted to ensure the safety of the care recipient. However, they felt that an indication of a low or high confidence percentage might help formal caregivers to organize their care better. For example, they can prioritize their visits depending on the system’s reliability percentages: 

For me, it’s fine to read that information, whether it is 50 percent or 80 percent, that doesn’t matter. But I think for professional caregivers that it does matter, because if they receive 6 notifications and one has 30 percent reliability and the other 80 percent. Then they will first go to the one with 80 percent reliability.

Participant 4

Interestingly, 1 informal caregiver expressed that viewing percentages might be slightly confusing for them to interpret; thus, simple terms such as “very reliable” or “less reliable” can be used:

Now I can’t judge 10 percent or 80 percent or 20 percent or whether that’s right.

Participant 3

Furthermore, informal caregivers demonstrated a willingness to offer feedback to the system to enhance the reliability of the system’s alerts. However, it was recognized that this responsibility should be shared with other caregivers, particularly formal caregivers, who are also involved in responding to alerts and, thus, can also provide context-aware and detailed feedback to enhance the system’s learning:

The system is self-learning, so I’m actually positive about it. I hope people understand that when they provide feedback, they need to specify what exactly went wrong, so that the system can learn from that. For example, if someone didn’t fall but just lay down on the couch, then this should be adjusted. The system can become smarter by processing more data and thus increase the reliability of notifications. So, it’s important to add more context in order for the system to learn from it.

Participant 5

##### Verifiability: Possibility to View the Electronic Health Record and Connect With Formal Caregivers

The possibility to view the electronic health records and connect with the concerned formal caregivers was found to be very handy and desirable. These functionalities also support the notion of an all-inclusive platform:

For example, If I want to speak to Mrs. Baker [formal caregivers], I click on Mrs. Baker and she can guide me further.

Participant 1

I think it is always desirable to have everything in one place and not have to deal with various different systems again, also considering different passwords and identification or authentication as well.

Participant 2

#### Mixed Experiences

##### Reminder: Receiving a Reminder in Case of a Missed Emergency Call

Mixed experiences were reported regarding the reminder that was received in case of a missed emergency call. Some informal caregivers found it useful, and others thought that reminders were not necessary for them, as this would be more useful for formal caregivers, depending on their personal situation. One informal caregiver experienced the reminder as confronting:

I would like a care professional to receive such a reminder when she falls, because I am always at a distance.

Participant 1

If I see this message, and realize I’ve missed the emergency call, then I feel like I should have been more attentive, then I would like to have the information quickly and in a concise format, without having to read through a lot of details.

Participant 3

##### Suggestions: Receiving Suggestions Regarding What Actions to Take

The use of suggestions along with alerts or notifications was found to be debatable in older adult care. On one hand, informal caregivers found suggestions to be valuable in situations such as emergencies, where they panic or are unsure about the possible actions to take to facilitate the right care: 

We all know what stress and panic can do, in those moments we can sometimes make stupid decisions, or forget the best order of doing things. So, having such a suggestion can serve as a helpful guide.

Participant 4

I feel that falling is different from agitated behavior. Falling means immediate danger, while agitated behavior often arises in the context of the dementia process that people experience. In such cases, it would be helpful to receive tips on what to do.

Participant 1

On the other hand, some informal caregivers felt that suggestion were unnecessary and subjective to the care experiences of the informal caregivers:

I think many people would appreciate it. You see, I’ve been working in healthcare for many years, so I’m familiar with these things. I believe there are many people who would benefit from receiving suggestions on what to do in certain situations. While I may quickly come up with solutions based on my experience, this is not the case for everyone. Thus I think many people would find it supportive.

Participant 1

Moreover, informal caregivers expressed concern that if suggestions are system generated, they will be generic, which could potentially limit their thinking to the provided suggestions only, thus losing the personal touch in care and inducing the feeling of annoyance: 

I find this terrible, very annoying. Because I’m already stressed out, and then I get those too obvious suggestions that say “do this, do that.” My stress levels are already high and then I read something stupid...no thank you. Very irritating...

Participant 2

Overall, while the usefulness of suggestions differs from person to person, it would be valuable to have such an option for those who are willing to receive it.

##### Social Learning: Reading Other Informal Caregiver’s Experiences

The informal caregivers reported varying experiences regarding the page that included stories about the experiences of other informal caregivers. Some indicated this was valuable for them because reading about the experiences of others could provide them with some support, insight, and inspiration regarding how others handled certain situations:

You can share your experiences, this is not strictly necessary, but it does help because then you realize you’re not the only caregiver. And when you share experiences, you get tips and tricks, you can learn from them. I think this is really great.

Participant 5

Other informal caregivers who are experienced (either caring for a long time or were medical or care professionals) or have support from other informal caregivers did not perceive social learning as advantageous. However, they seek value in social learning for people who are providing care by themselves and do not have a social network to support them:

I don’t need this, because I actually know the possibilities in the field quite well and I experience a lot of support from my brother and sister. We are doing well together...

Participant 1

I think that for some people who live alone and are the only informal caregivers, it would be a welcome thought. This is about how you have organized your caregiving network. That is not always easy, sometimes quite complicated. So in that sense, it could be a very helpful feature.

Participant 3

#### Suggestions for Improving the User Interface Prototype

##### Overview

Overall, the conceptual workflow of the prototype of the interaction platform was assessed positively by the informal caregivers. They indicated that most of the screens were clear and understandable. However, they provided some suggestions about screens or connections that were perceived as less logical or where improvements could be made.

##### Improvements in Conceptual Flow

Informal caregivers highlighted that some choices regarding the notification settings were repetitive or unnecessary, which made the flow unclear or redundant. Specifically, in Figure 3 (third screen [agitation] and fourth screen [fall incident]), the option to share the respective information with the formal caregiver was given; however, informal caregivers already mentioned their choices to share or not share the information with formal caregivers while adjusting their preference (Figure 2). Consistent with an iterative process, this was adjusted for the next (last 2) interview sessions:

Here, I again have the choice if I want to share with a care professional. But if that happens again, then I wonder if I have set it up correctly in the settings. So does this still appear on my screen? In the beginning, you make a choice about sharing information with a care professional, and here that comes up again, so it’s kind of redundant.

Participant 3

This is what I don’t understand. If I let the notifications go to the home care professionals for this situation, then I should not have to fill this in [choice for the content of the notification].

Participant 4

Furthermore, informal caregivers indicated that it was inconvenient to immediately receive the option to provide feedback to the system in case of an emergency notification as they mentioned, at that moment, they were not thinking about the feedback and were probably not the right person to provide the information. It was suggested to send a reminder to provide feedback at a later moment. Moreover, there should be an option to receive more details about the situation:

Provide feedback on this notification, yes that can be useful, but it has to be at a later moment. You don’t do this in the notification itself, but you can add at a later moment what the issue was and whether the notification was accurate.

Participant 2

For this, I would appreciate a reminder. I don’t necessarily enjoy receiving a lot of notifications all the time, but specifically for this purpose, yes. It’s about helping each other and helping the system learn, and thus improving the care. And I think when I’m actually there [at loved one] or when I come from there, then I might forget that. So, a reminder would be helpful, but it would be good to have a choice in the type of notification.

Participant 3

Informal caregivers also suggested that feedback should be provided to them after they received a notification, so that they know that someone handled the situation and what actions have been taken:

I think that is a bit of a gray area, so you received or made a notification, but what happens with it? That I would expect to receive feedback on.

Participant 5

Now I still have the feeling like I have to go there because I don’t know if it [notification] has been received and if someone is going there.

Participant 1

##### Improvements in Visual Design

The informal caregivers suggested to include a clear visual indication when a deviation in behavior was noticed by the system, for example, a warning sign. In addition, an informal caregiver mentioned it would be more useful to express reliability in words instead of percentages, as this might be easier to interpret. Finally, informal caregivers suggested that the prototype could be improved by providing information in a more visual manner and including more graphs, images, and pictograms, as this could make it easier to interpret the information they were looking for:

At a glance, I can see that everything is going well...but then [in case of deviation] could have a different color like red, and for yourself there could an exclamation mark or warning sign to indicate that this is not optional information but something that needs to be looked into because it is not as it should be.

Participant 5

## Discussion

### Principal Findings

In this study, informal caregivers showed a significantly positive attitude toward using USSs driven by AI algorithms for providing care to home-dwelling older adults with cognitive impairment. However, a previous study that explored care recipients’ perspectives regarding AI in health care revealed hesitancy, primarily driven by worries related to safety, privacy, and autonomy [29]. This divergence could be attributed to 2 factors: the difference in the study population and the potential lack of knowledge about USSs (in general, technological care solutions) among the previous study’s participants. The previous study by Richardson et al [29] focused on care recipients’ perspectives, whereas this study involved informal caregivers who might have a different perspective, as USSs will be monitoring the care recipient and it does not concern informal caregivers. Furthermore, many people have limited knowledge about AI algorithms and view AI as a “black box” [30]. Previous studies suggest that educating and engaging individuals about AI can enhance their trust in AI and contribute toward its successful implementation in health care [29,30]. In this study, USSs were explained using a video prototype and additional verbal explanations, which most likely increased the participants’ awareness of AI use. However, individual differences in understanding AI might also influence their positive and accepting attitude toward USSs.

USSs rely on AI algorithms to predict the behavior of older adults with cognitive impairment, which might not function flawlessly and could misclassify certain behavior patterns. Therefore, care providers need to be cautious and should not become overly reliant on USSs, as it may lead to incorrect care choices [31]. This would present an ethical issue regarding accountability, as it prompts the question of who is responsible and to what extent [31]. Educating caregivers about its use, capabilities, and limitations might help to overcome this risk. In addition, the risk of bias when using AI in health care should also be considered [31,32]. If the training data predominantly represent a specific population (sex, age, ethnicity, etc), they might create biases [32]. This risk could be mitigated by ensuring representative and inclusive training data sets, that is, including data from a wide range of individuals with different demographic characteristics and backgrounds when training the AI algorithm [32].

Furthermore, the informal caregivers recognize the value of USSs as a supportive tool in the care of home-dwelling older adults with cognitive impairment [2]. Specifically, USSs can facilitate appropriate care decision-making, contributing to their peace of mind while also creating a safe environment for their care recipients. However, they also expressed some concerns regarding the possible information overload, substitution of the human aspect in care provision, and overinterpretation of data. To mitigate these issues, they acknowledged the importance of setting up (make agreements about the monitored activities and strategies regarding communication) the solution together with other stakeholders (specifically, formal caregivers). This is consistent with the CeHRes road map, that is, for successful implementation of an eHealth technology, it is important to consider the perspectives and needs of the different stakeholders involved [15,33]. Moreover, it can be said that by combining the strengths of technology with the insights and expertise of caregivers, a more comprehensive and effective care approach and implementation could be achieved.

In addition, informal caregivers experienced the lo-fi prototype and the use of most PSD features elicited in a previous study as positive [7]. Particularly, participants valued the possibility to personalize the settings and change them to their preferences at any given moment in time. The use of the personalization feature, as suggested in the PSD theory, has the potential to enhance the usefulness of eHealth technologies such as USSs [7]. However, before making personalized solutions, the personalization-privacy paradox should be considered carefully [34]. This paradox demands right balance between offering personalized experiences and safeguarding user privacy [34,35]. This balance might be achieved by implementing robust privacy measures, obtaining informed consent, being transparent about data use, and providing users with control over their data as described in the ethical guidelines issued by the European Commission for trustworthy AI [36] and the European Health Data Space regulation [37]. Moreover, regarding the PSD features reminders, suggestions, and social learning, the experiences were slightly mixed. These findings emphasize the importance of a user-centered design approach, as the preferences of each individual can vary depending on the care situation, personal circumstances, and preferences in IC [15]. For example, the travel distance to the care recipient could influence their choice regarding whether they want to receive an emergency call when a fall incident occurred.

### Implications for Future Studies and Practices

For the successful implementation of a complex eHealth intervention such as USSs for home-dwelling older adults with cognitive impairment, a holistic design approach is required [15]. It is important to consider the perspective of different key stakeholders such as informal and professional caregivers, care recipients, and care organizations and secondary stakeholders such as health insurers, governments, and technology businesses while designing and implementing such solutions. In future studies, it would be interesting to perform the next design iteration by using the results of this study as a starting point. Gradually, a high-fidelity prototype of the user interface could be created and evaluated with different stakeholders in the care of older adults with cognitive impairment. Furthermore, the creation of personas would be helpful in studies, as different types of end users may have different needs and requirements [38]. The personas could be based on characteristics such as caregiving experience, educational level, or need for cognition [38]. In addition, it would be interesting to explore the ethical implications of implementing a smart monitoring and communication system for home-dwelling older adults, which could be performed using in-depth interviews with involved stakeholders.

Before the implementation stage, a business modeling approach can be used. It provides insight into how value is generated and delivered to customers, which should be considered to bring eHealth technology to the market [39]. In addition, the implementation of such a solution requires guidelines and agreements (about how to work with such a system) within organizations and at the government level. Caregivers should be educated about how to interact with the system and interpret and communicate the data. In addition, it is essential to consider regulations such as Medical Device Regulation to determine whether USSs will be categorized as medical devices. Medical Device Regulation offers provisions to address privacy and security concerns, especially regarding medical devices that collect and process personal health data [40]. Furthermore, as USSs use AI, it is crucial to take into account the new AI Act proposed by the European Union (EU). This act aims to regulate the use of AI in EU countries, ensuring better conditions for the development and use of AI technologies (EU AI Act, 2023) [41]. In this act, different rules will apply to different risk levels, with USSs probably falling under the category of high risk, and they will be subjected to a high degree of regulations [41]. This might have consequences for the extent and manner in which AI is applied.

### Strengths and Limitations

The key strength of this study is rooted in its methodology, specifically the adoption of a participatory development process that actively engaged informal caregivers during the early development stage of the interaction platform. The designed lo-fi prototype of the interaction platform provided an overview of the overall conceptual workflow and PSD features in different care scenarios to informal caregivers. While lo-fi prototyping is cost-effective, it can be seen as an opportunity for rapid development with end users in the early stages of development. Specifically, a task-based study design in conjunction with a think-aloud approach was used for this study. The task-based study design facilitated a realistic evaluation of user interactions with interfaces in various care scenarios [18,19], and the think-aloud approach provided direct access to users’ thoughts, perceptions, expectations, experiences, and decision-making processes during their interactions with the interfaces and PSD features [20]. By combining these 2 approaches, a comprehensive understanding of potential issues (in the used PSD features) and areas for improvement (in the conceptual workflow) was obtained, which could be incorporated into subsequent design iterations. In addition, the use of a video prototype assisted participants in comprehensively understanding the proposed USS. This, in turn, facilitated the development of more concrete and well-informed themes regarding the implementation outcomes and preconditions necessary for the successful integration of novel USSs.

Along with strengths, this study also has some limitations, which should be considered when interpreting the results. First, all the informal caregivers (6/6, 100%) who participated in this study had previous experience with technological interventions in care provision (eg, Caren platform) and had previously participated in related studies. While it is important to acknowledge that the findings of this study may not be fully generalizable to participants with no previous experience in digital care technology, the overall growth in digital literacy is noteworthy and holds promise for the realization of the study’s findings. Second, most informal caregivers (4/6, 67%) who participated in this study reported that their care recipient had received a formal diagnosis of cognitive impairment. However, some informal caregivers also expressed their own opinions regarding the indication of cognitive impairment in their care recipients. Given the scope of this study, which aimed to explore the perspectives of informal caregivers, their opinions hold high value in this study [42-44].

Third, it is important to note that this study was conducted in the Netherlands, which may limit the generalizability of the results to international health care infrastructure. Different countries have diverse regulations and policies regarding older adult care; thus, different expectations and preimplementation conditions regarding USSs can be imagined. Finally, it is worth mentioning that data saturation was not reached in this study, as new information was obtained from all interviews. This indicates that there may be additional themes that were not fully explored, suggesting that the results of this study may not be exhaustive. However, it is important to recognize that the design process was iterative, and during the evaluation of the lo-fi prototype, the aim was to further enrich the platform, making data saturation less critical for this stage of development [45].

### Conclusions

Overall, informal caregivers of older adults with cognitive impairment had positive expectations regarding the implementation of USSs. They expect the use of such a system to contribute to care decision-making and to provide insight into the situation of the care recipient. However, information overload and loss of human aspect were perceived as risks. To successfully implement a USS, good communication and agreements among informal caregivers, formal caregivers, and the care recipient are needed, thus necessitating a holistic approach in the development and implementation process. Informal caregivers were quite positive about the lo-fi prototype of the user interface and the application of PSD features; however, there were also mixed experiences and suggestions for improvement regarding the conceptual flow and visual design of the prototype. Personalization of the settings of the prototype was perceived as highly valuable. The results of this study, especially the identified concerns, should be considered in the further development and implementation of USSs for home-dwelling older adults with cognitive impairment.

## Appendix

Multimedia Appendix 1 The semistructured interview script used for qualitative data collection.

## Abbreviations

AI: artificial intelligence
CeHRes: Center for eHealth Research and Disease Management
CSI: channel state information
EU: European Union
IC: information communication
lo-fi: low-fidelity
PSD: persuasive system design
USS: unobtrusive sensing solution

## Data Availability

In this study, some personal details about the participants were obtained, constraining our ability to make quantitative data available for any secondary analysis. However, upon request, a summary of the qualitative data (after anonymization) can be provided.
