# Supplementary material for: Low-Fidelity Prototype of a Sensor-Dependent Interaction Platform: Formative Evaluation With Informal Caregivers of Older Adults With Cognitive Impairment
Source: JMIR Form Res. 2024 Mar 22;8:e53402. doi: 10.2196/53402 (PMC10998178; doi:10.2196/53402)
Supplement: Multimedia Appendix 1 [file formative_v8i1e53402_app1.pdf]

Interview guide for interviews with informal caregivers

Introductie
Ik zal beginnen met mijzelf voor te stellen: Ik ben Karen en studeer Health Sciences aan de Universiteit van Twente. Ik ben momenteel bezig met mijn scriptie onderzoek, vandaar dat u bent uitgenodigd voor dit gesprek.

Uitleg doel onderzoek en procedure
Het doel van dit onderzoek is te achterhalen hoe professionele zorgverleners en mantelzorgers denken over een smart monitor systeem om thuiswonende ouderen met dementie te monitoren. Het interview zal bestaan uit twee delen. Eerst zal ik een video laten zien waarin de smart monitor systeem wordt uitgelegd en zal ik u hier wat vragen over stellen. Het is de bedoeling dat aan dit systeem een communicatieplatform gekoppeld wordt, wat informatie over de situatie van uw naaste doorgeeft aan u als mantelzorger of een professionele zorgverlener. Daarom zal ik u vragen om een prototype voor een communicatieplatform uit te testen en heb ik ook hierbij wat vragen.

Vertrouwelijkheid en data
Alle informatie die u met mij deelt zal vertrouwelijk behandeld worden, dit betekent dat het niet terug te leiden zal zijn naar u als persoon. Gegevens worden veilig opgeslagen en zullen niet met andere partijen worden gedeeld. Gegevens zullen na afronding van het onderzoek worden verwijderd.

Audio opname
Ik zou graag een audio opname willen maken van het interview als u hiermee instemt. De audio opname wordt alleen gebruikt voor data-analyse doeleinden.

Duur van interview en vroegtijdig stoppen
De totale duur van het interview zal ongeveer 1 tot maximaal 1.5 uur zijn. Mocht u tijdens het interview een pauze willen of vroegtijdig willen stoppen dan is dit geen probleem en mag u dit gerust aangeven. Dit mag op elk moment tijdens het gesprek en u hoeft hier geen reden voor te geven.

Toestemming
Gaat u nog steeds akkoord met deelname aan het interview? Dan zou ik willen vragen dit formulier te ondertekenen.
Online interview: mondeling informed consent opgenomen door audio-recorders
In-person interview: geschreven informed consent formulier

Ondertekenen informed consent formulier
Opname apparatuur aanzetten

Achtergrond informatie
1. Geslacht: man/vrouw
2. Leeftijd van zorgvrager
3. Leeftijd mantelzorger
4. Aantal jaren mantelzorger:
5. Schatting aantal uren/week aan mantelzorgtaken
6. Welke mantelzorgtaken
7. Relatie tot zorgvrager
8. Diagnose zorgvrager
    a. Wanneer zijn de symptomen begonnen
9. Waar/hoe woont de zorgvrager
    a. Hoe ver bij mantelzorger vandaan?
10. Primaire mantelzorger?
    a. Andere mantelzorgers betrokken?

11. Aantal professionele zorgverleners betrokken
12. Gebruik van andere technologieën bij de zorgverlening?
    a. Monitor technologie
    b. Communicatie technologie (platform Caren)

Video aanzetten en kijken

Onduidelijkheden
U heeft een video gezien waarin het smart monitor systeem wordt uitgelegd. Het systeem kan dus door gebruik te maken van Wi-Fi signalen, door de muren heen registreren welke activiteiten uw naaste uitvoert. Vervolgens kunt u en andere zorgverleners deze informatie binnenkrijgen op een app op uw telefoon. Zijn er naar aanleiding van deze video op dit moment nog vragen of onduidelijkheden over het smart monitor systeem?

Interviewvragen m.b.t. smart monitor systeem
Algemene indruk
1. Wat is uw algemene indruk van het smart monitor systeem zoals het is uitgelegd in het video prototype?
2. Hoe denkt u erover dat dit systeem op ieder moment plekken/activiteiten kan registreren?

Uitleg usability test
Dan gaan we nu verder met het testen van het prototype voor het communicatie platform. Het prototype is echt pas een eerste versie, dus echt alleen de basis van het communicatie platform. Ik heb u een link gestuurd waar u het prototype kan bekijken en door kan klikken. Het prototype bestaat uit een aantal schermpjes die u als mantelzorger in een app op uw telefoon zou kunnen ontvangen. Ik wil u straks vragen om deze schermpjes zo te behandelen alsof het een echte app zou zijn. Ik zal u een situatie voorleggen met een opdracht die u mag uitvoeren. U kunt hierbij geen fouten maken. Terwijl u dat doet, wil ik u vragen om alles wat u doet en denkt hardop te benoemen en ook eventuele suggesties te benoemen. Wij willen graag weten hoe we het systeem moeten aankleden en wat u nodig heeft in het systeem. Er is hierbij dus geen goed of fout, wij zijn enkel geïnteresseerd in uw ervaring en uw mening. U mag dus zeggen wat u wilt terwijl u de opdrachten uitvoert.

Usability test
Opdracht 1: login en voorkeuren aanpassen (tunneling and personalization)
Als eerste wil ik u vragen om in te loggen in de app en uw voorkeuren met betrekking tot het ontvangen van meldingen over acute / risicovolle situaties aan te geven.
1. Hoe denkt u over de mogelijkheden om uw voorkeuren aan te passen?
2. Wat vindt u van de manier waarop u door de schermpjes wordt geleid?

Opdracht 2: vallen (suggestion, reduction and tailoring)
Ik wil u nu graag een situatie voorleggen waarin uw naaste is gevallen. U ontvangt een telefoontje van het smart monitor systeem. U heeft het telefoontje niet beantwoord, dus het systeem stuurt u na 5 minuten een herinneringsmelding.
1. Wat is uw mening over het ontvangen van een direct telefoontje in een nood/acute situatie zoals een valincident? En wat vindt u ervan dat u daarna een melding krijgt in het geval dat u niet binnen 5 minuten reageert?
2. Denkt u dat het meerwaarde heeft/gunstig is om direct professionele zorgverleners op de hoogte te brengen?
3. Wat vindt u ervan dat het systeem u informatie geeft over de betrouwbaarheid van de gegevens en suggesties geeft voor welke acties u zou kunnen ondernemen?
4. Wat vindt u ervan om aan het systeem terug te koppelen in hoeverre de melding accuraat was (bv. valse alarm)?
5. Was er iets dat anders ging dan wat u had verwacht?

Opdracht 3: geagiteerd gedrag (reduction, suggestion, tailoring)

Ik wil u vragen om een situatie voor te stellen dat uw naaste de laatste tijd steeds vaker geïrriteerd of boos is. Ik wil u vragen om door de schermpjes te klikken en meer informatie op te vragen over dit gedrag van uw naaste.

1. Hoe denkt u over het krijgen van het gedetailleerde overzicht en de suggesties die het platform doet in deze situatie?
2. Wat vindt u van de mogelijkheid om deze informatie met een professionele zorgverlener te delen?
3. Wat had u van het systeem verwacht/willen zien in deze situatie?

Opdracht 4: zelfzorg activiteiten (personalization)
Er zijn ook bepaalde activiteiten/gedragingen die behoren tot 'zelfzorg'. Dit kan bijvoorbeeld gaan om het onjuist innemen van medicatie, of dit compleet vergeten, of om eten, drinken en slaapgedrag. Ik wil u vragen om door de schermpjes te klikken en uw voorkeuren zo aan te passen dat u een melding krijgt van activiteiten/gedrag waar u zich zorgen om maakt als het om uw naaste gaat.

1. Wat denkt u van de mogelijkheden tot personalisatie (het aanpassen van uw voorkeuren) bij de zelfzorg activiteiten?
2. Wilt u hier nog wat aan toe voegen?

Opdracht 5: algemene systeem features – langdurig monitoren en social learning (social learning)
Zodra u de app opent komt u op het home-screen waar u een overzicht kan vinden van de huidige situatie van uw naaste en ook andere functionaliteiten van het systeem kan bekijken. Ik wil u vragen om het home-screen te bekijken en uit te proberen welke functies/pagina's het heeft.

1. Zou het voor u motiverend werken om ervaringen van andere mantelzorgers te lezen? Waarom wel/niet?
2. Wat vindt u van de mogelijkheid om over een langere periode te monitoren en hier een rapport/overzicht van te kunnen ontvangen?
3. Is er iets wat u nog wilt toevoegen?

Interview vragen m.b.t. user interface

1. Denkt u dat het makkelijk zou zijn om het systeem te gebruiken?
2. Zou u bereid zijn om een eigen bijdrage te betalen voor het gebruik van een dergelijk systeem?
3. Wat zijn volgens u goede en minder goede punten aan het prototype voor het communicatie platform?
    a. Hoe zou dit verbeterd kunnen worden?
4. Wat mist er volgens u nog aan de schermpjes van het communicatieplatform/prototype?
5. Welke pagina's/functies vindt u overbodig of minder noodzakelijk?
6. Wat verwacht u verder nog van dit platform?
7. Zou u dit platform graag geïntegreerd zien met andere zorgplatforms zoals Caren?

Intention to use

1. Welke activiteiten moet het systeem volgens u sowieso kunnen registreren?
    a. Nood/acute situaties
    b. Zelfzorg activiteiten
    c. Waarom vindt u dit belangrijk?
2. Aan welke randvoorwaarden moet het systeem voldoen voor u om vertrouwen te hebben in het systeem?
    a. Waar moet het systeem voor u aan voldoen om veiligheid te garanderen?
    b. Waar moet het systeem aan voldoen om privacy te garanderen?

Afsluiting
Dan zijn we nu aan het einde gekomen van het interview. Zijn er nog dingen die u kwijt wil die nog niet aan bod zijn gekomen?
Zoals gezegd zullen gegevens vertrouwelijk worden behandeld en niet worden gedeeld met anderen. Ik zal nu het interview uittypen en de gegevens gaan analyseren. Deze informatie zal worden gebruikt om het systeem te verbeteren en zo in te richten zodat de eindgebruikers er wat aan hebben.

Ik wil u bedanken voor deelname aan het onderzoek. Mocht u achteraf toch nog vragen hebben dan kunt u mij altijd bereiken via de mail of telefonisch. U krijgt binnenkort een bol.com waardebon toegestuurd via de mail als bedankje voor uw deelname.

Einde formatieve evaluatie
Opname apparatuur afsluiten
